# Productive, metabolic and economic responses of Nellore young bulls fed diets with different non-protein nitrogen source combinations

**DOI:** 10.1007/s11250-026-05264-7

**Published:** 2026-07-30

**Authors:** Luiz Antonio Rodrigues, Priscilla Dutra Teixeira Borges, Camila Celeste Brandão Ferreira Ítavo, Gelson dos Santos Difante, Alexandre Menezes Dias, Vanessa Zirondi Longhini, Manoel Gustavo Paranhos da Silva, Lívia do Nascimento Gomes, Adrianni Dias Borges, Hevelyn de Almeida Cunha, Luís Carlos Vinhas Ítavo

**Affiliations:** Faculdade de Medicina Veterinária e Zootecnia (FAMEZ), Universidade Federal de Mato Grosso do Sul (UFMS), Av. Senador Filinto Müller, 2443, Cidade Universitária, Campo Grande-MS, 79070-900 Brazil

**Keywords:** Cinnamon, Weight gain, Garlic, Nitrogen source

## Abstract

Nutrition is a key determinant of performance, carcass traits, and production efficiency in beef cattle. In this context, feed additives have emerged as a promising strategy to enhance nutrient utilization and reduce production costs. We aimed to evaluate the effects of replacing urea with a product extruded urea enriched with essential oils (NFeed^®^) on nutrient intake, performance, blood parameters, and feed cost in feedlot-finished Nellore young bulls. Twenty-four non-castrated animals with an average initial body weight of 394.6 ± 53 kg were randomly assigned in a completely randomized design, following the treatments considering different combinations of NPN source in diet: 0% NFeed (100% of non-protein nitrogen from urea), 50% NFeed (50% of non-protein nitrogen from urea and 50% of non-protein nitrogen from NFeed), 75% NFeed (25% of non-protein nitrogen from urea and 75% of non-protein nitrogen from NFeed) and 100% NFeed (100% of non-protein nitrogen from NFeed). Final body weight, total weight gain, and average daily gain had a linear response, with the highest values in the 100% NFeed treatment. Feed efficiency and feed conversion exhibited linear effects, with the 100% NFeed diet resulting in the greatest feed efficiency and the lowest feed conversion. A quadratic effect was observed for plasma urea, and gamma-glutamyl transferase levels. The replacement of conventional urea with extruded urea enriched with essential oils (NFeed) represents a viable nutritional strategy for finishing beef cattle in feedlot systems. Increasing levels of NFeed linearly improved animal performance while maintaining similar feed intake, increasing feed efficiency reducing feed conversion, and the cost per kilogram of body weight gain. Thus, we recommend a partial replacement of urea by NFeed as a non-protein source in beef cattle feedlot.

## Introduction

The intensification of beef cattle production systems, especially under feedlot conditions, requires nutritionally balanced diets to optimize animal performance and economic returns. Urea, a widely used non-protein nitrogen source, balances nitrogen in diets with different energy levels, thereby improving productivity and reducing costs (Tedeschi et al. 2002). However, caution is needed because the rapid hydrolysis of urea in the rumen can lead to increased blood ammonia levels. This raises the risk of ammonia toxicity, resulting in poor performance and health outcomes in ruminants (Salami et al. 2020). In this context, novel technologies have been used to produce slow-release urea sources in the rumen, improving microbial utilization and lowering the risk of ammonia toxicity, such as extruded urea (Kozerski et al. 2021a). According to Kozerski et al. (2021b), the extrusion of urea with corn to make extruded urea changes the physical structure of the mixture, enhances its quality through starch gelatinization and complex formation with urea crystals, and reduces the rapid solubilization of nitrogen in the rumen.

In addition to synchronizing rumen ammonia production with the availability of fermentable energy, feed additives are essential for improving animal performance and rumen health. Natural feed additives, including secondary plant metabolites such as saponins, tannins, flavonoids, and essential oils, have shown promise in modulating rumen fermentation (Cobellis et al. 2016).

Essential oils have gained attention as natural feed additives that can enhance animal performance in a sustainable way. Their antimicrobial properties allow for modulation of rumen microbial populations, including ammonia-producing bacteria, influencing the utilization of nutrients (Torres et al. 2021). When used at the appropriate dosage, these secondary plant compounds can improve rumen fermentation, reduce nitrogen and energy losses, and promote growth in ruminants (Ferreira et al. 2024). Some essential oils, particularly those derived from garlic and cinnamon, have been shown to modulate rumen microbiota, increase propionate production, lower the molar proportion of acetate, boost nitrogen retention, and reduce methane emissions (Busquet et al. 2005; Besharati et al. 2023; García-Rodríguez et al. 2024).

Recent results have demonstrated the superiority of extruded urea as an isolated non-protein nitrogen source, and similarly, the inclusion of essential oils in ruminant diets has shown effectiveness in ruminal modulation. However, we hypothesized that the increased levels of NFeed in replacement of urea would enhance intake, productive performance, and without to affect metabolic parameters in beef cattle during the finishing phase. We aimed to assess the effects of substituting livestock urea with extruded urea with essential oils (NFeed) on nutrient intake, productive and economic performance, and blood parameters of Nellore young bulls finished in a feedlot.

## Materials and methods

The experiment was conducted in the Feedlot sector of the School Farm of the Federal University of Mato Grosso do Sul, located in the municipality of Terenos, Mato Grosso do Sul, Brazil. This study was carried out following recommendations of Animals (Scientific Procedures) Act 1986 and EU Directive 2010/63 guidelines for animal experiments and approved by the Ethics Committee on the Use of Animals of the Federal University of Mato Grosso do Sul (Protocol 1216/2022).

### Experimental design, animals, and diets

Twenty-four Nellore young bulls (non-castrated animals) from the school farm herd, with an average initial body weight of 394.60 ± 53 kg and 24 months of age were housed in individual pens. The study was carried out as a completely randomized design with four treatments and six replicates per treatment. The treatments consisted of different combinations of non-protein nitrogen sources through the replacement of urea with extruded urea enriched with garlic and cinnamon essential oils (NFeed). Four treatments were formuled: (1) 0% NFeed (100% of non-protein nitrogen from urea), (2) 50% NFeed (50% of non-protein nitrogen from urea and 50% of non-protein nitrogen from NFeed), (3) 75% NFeed (25% of non-protein nitrogen from urea and 75% of non-protein nitrogen from NFeed) and (4) 100% NFeed (100% of non-protein nitrogen from NFeed).

The chemical composition of ingredients used to formulation of diets are presented in Table 1. The concentrate contained ground corn, soybean meal, livestock urea, and NFeed^®^ (Pajoara Indústria. e Comércio Ltda, Campo Grande-MS, Brazil). Non-protein nitrogen sources were incorporated into the diets to supply roughly one-third of the total nitrogen intake, representing 31.6% of the dietary crude protein (CP), equivalent to 42.1 g/kg of CP from NPN sources. NFeed^®^ is composed of extruded urea (livestock urea, ground corn grain, and sulfur flower in the proportions of 70%, 26.8%, and 3.2%; Kozerski et al. 2021b); enriched with garlic and cinnamon essential oils (430 mg/Kg of cinnamaldehyde and 70 mg/kg of diallyl; Oliveira et al. 2025). Cinnamaldehyde and diallyl are active ingredients belonging to cinnamon and garlic, respectively.

**Table 1:** Chemical composition (g/kg) of ingredients in experimental diets

	DM (g/kg)	OM	CP	NDF	EE	TDN
		g/Kg DM				
Whole plant corn silage	310.2	947.1	83.8	521.5	27.7	648.6
Ground corn	887.1	984.0	90.2	197.4	40.0	872.8
Soybean meal	886.5	933.9	488.5	147.9	19.3	802.7
NFeed^#^	912.3	958.0	2029.7	36.2	31.7	223.0
Urea	979.0	997.5	2818.7	-	-	-
Mineral mix	990.0	-	-	-	-	-

^#^ Extruded urea enriched with essential oils

The diets (Table 2) were offered *ad libitum* once daily during the morning (0900 h). Diets were formulated to have 33.5% corn silage and 66.5% concentrate diet. The experimental period lasted 100 days, including 14 days of adaptation to the facilities and diets. During this adaptation phase, animals received the experimental diet with feed initially restricted to 50% on the first day, followed by a daily increase of 5% until reaching the target intake. At the start of the adaptation period, all animals were treated for internal and external parasites using a dewormer (Cydectin; Zoetis Veterinary Medicine Industry Ltda., Campinas, SP, Brazil) and were vaccinated against clostridial diseases (Excell 10; Dechra Brasil Veterinary Products Ltda., Londrina, PR, Brazil) and respiratory diseases (Inforce 3; Zoetis Veterinary Medicine Industry Ltda., Campinas, SP, Brazil).

**Table 2:** Ingredients and chemical composition of experimental diets with different levels of NFeed (DM basis)

	NFeed levels			
	0% NFeed	50% NFeed	75% NFeed	100% NFeed
Ingredients, g/Kg				
Whole plant corn silage	335.0	335.0	335.0	335.0
Ground corn	600.0	597.0	595.4	593.9
Soybean meal	30.0	30.0	30.0	30.0
Mineral mix^a^	20.0	20.0	20.0	20.0
NFeed^#^	-	10.5	15.8	21.1
Urea	15.0	7.5	3.8	-
Chemical composition, g/Kg DM				
Dry matter (g/kg)	697.26	696.83	696.63	696.41
Organic matter	948.93	948.04	949.21	950.38
Crude protein	159.34	157.30	156.59	155.50
Equivalent CP from NPN source	59.36	57.60	57.05	56.10
Neutral detergent fiber	235.08	234.47	234.14	233.83
Ethereal extract	36.30	36.17	36.10	36.03
Total digestible nutrients	799.88	797.03	795.49	794.07

0% NFeed (100% of non-protein nitrogen from urea), 50% NFeed (50% of non-protein nitrogen from urea and 50% of non-protein nitrogen from NFeed), 75% NFeed (25% of non-protein nitrogen from urea and 75% of non-protein nitrogen from NFeed) and 100% NFeed (100% of non-protein nitrogen from NFeed)

^#^ Extruded urea enriched with essential oils

^a^Assurance levels per kilogram of product: 80 g/kg P, 200 g/kg Ca, 123 g/kg Na, 2000 mg/kg Zn, 1000 mg/kg Mn, 400 mg/kg Cu, 50 mg/kg I, 50 mg/kg Co, and 5 mg/kg Se

### Nutrients intake and performance

The feed offered was recorded and weighed daily, and orts were collected and weighed the following morning. Intake of dry matter (DMI) and nutrients was estimated by subtracting the nutrient content of the orts from the total nutrient supply in the offered diet.

Weekly samples of the feed, orts, and diet ingredients were obtained throughout the experimental period. The weekly subsamples for each material were combined to generate a representative composite sample. The samples were pre-dried in a forced ventilation oven at 55 °C for 72 h, and ground in a grinder with a 1-mm mesh sieve. Chemical analyses were performed according to INCT-CA (Detmann et al. 2021).

The animals were individually weighed at the beginning and the end of the experimental period after fasting for 16 h to measure total weight gain and average daily gain (ADG). The feed efficiency was calculated as the ratio of ADG and DMI and inversely calculated as the feed conversion ratio.

### Blood analyses

Blood collection was carried out before slaughter (day 97). Samples were obtained via puncture of the coccygeal vein using vacutainer tubes containing EDTA. The tubes were centrifuged at 2,000 × g for 10 min, and the resulting plasma was separated and stored at − 20 °C until analysis. Biochemical analyses were conducted at the Clinical Analysis Laboratory of the Faculty of Veterinary Medicine and Animal Science – UFMS.

The evaluated parameters included total protein (kit ref. 04657586), albumin (kit ref. 04657357), creatinine (kit ref. 10886874), cholesterol (kit ref. 10745065), triglycerides (kit ref. 04657594), glucose (kit ref. 04657527), urea (kit ref. 11200666), alkaline phosphatase (kit ref. 04657373), aspartate aminotransferase (AST; kit ref. 10745120), and gamma-glutamyltransferase (GGT; kit ref. 05401461). All analyses were performed using commercial kits (Roche Diagnóstica, São Paulo, Brazil) according to the manufacturer’s instructions on a Cobas c 111 analyzer (Roche Diagnóstica Brasil, São Paulo, Brazil).

### Slaughter and carcass assessment

Nellore young bulls were slaughtered at a commercial slaughterhouse following the rules established by the Regulations for the Industrial and Sanitary Inspection of Products of Animal Origin—RIISPOA (2017). Animals were slaughtered via concussion followed by jugular vein exsanguination. Subsequently, hides were removed, and evisceration was performed. After evisceration, carcasses were immediately weighed to determine hot carcass weight (HCW). Carcass yield (CY) was calculated following the methodology described by Gomes et al. (2021).

### Economic performance

The economic analysis was based on the market value of silage and concentrate before the experiment began, as well as the arroba (15 kg of carcass equivalent) at slaughter time (US$ 42.76). Revenue per animal (US$) was calculated by multiplying the hot carcass weight (HCW) by the market value per kg of carcass equivalent. The margin was determined by subtracting the total feed costs from the revenue. All results are presented in US$/animal.

### Statistical analysis

The data were submitted to analysis of variance using the PROC GLIMMIX procedure of SAS (SAS University Edition, SAS Institute Inc., Cary, CA, USA), following model below:

$$\>{\rm{Yij = \mu + Ni + \varepsilon ij}}$$

Where: Yij: value observed at the different levels of replacement of livestock urea for extruded urea enriched with garlic and cinnamon essential oils (NFeed) i, in repetition j; µ = overall mean effect; Ni: effect of different levels of replacement of livestock urea for extruded urea enriched with garlic and cinnamon essential oils (NFeed) (i = 0%, 50%, 75% e 100%); εij: random error, associated with each observation i and j.

The effect of NFeed levels was analyzed using the PROC REG procedure of SAS (SAS University Edition, SAS Institute Inc., Cary, CA, USA) by first-degree regression: yij = β0 + β1*x + εij, and second-degree: yij = β0 + β1*x + β2*x2 + εij; where Yij: value observed value; β0, β1, and β2: equation parameters; x: replacement of livestock urea for NFeed levels; εij: random error, associated with each observed value i and j.

The means per treatment were compared using the Tukey test. Statistical significance was declared at *P* ≤ 0.05 and tendencies were defined as 0.05 < *P* ≤ 0.10. Pearson correlation coefficients between blood parameters and animal performance were estimated using the PROC CORR procedure in SAS (SAS University Edition, SAS Institute Inc. Cary, CA, USA).

TWG and ADG were analyzed using the PROC MIXED in SAS (Statistical Analysis System, version 9.2), through the model:

$${Y_{ijt}} = \mu + {N_i} + {\rm{ }}{{\rm{d}}_j}_{(i)} + {\rm{ }}{{\rm{P}}_t} + {\left( {NP} \right)_{it}} + {{\rm{e}}_{ijt}}$$

where: *Y*_*ijt*_ are the observed values, µ is the overall mean, *N*_*i*_ is the *i*th level of the fixed effect of NFeed levels, d_*j(i*)_ is the random effect of the jth young bull within the ith NFeed level, *P*_*t*_ is the *t*th level of the fixed effect the period, and *e*_ijt_ is the random error associated with *Y*_*ijt*_.

The covariance structure with the smallest Bayesian information criterion (BIC) and finite sample corrected Akaike information criterion (AICC) was used in the analysis. Treatments were compared using Tukey test. The SLICE function of SAS was used to determine simple effects within period.

## Results

The treatments had no effect on dry matter intake (DMI) or the intake of other nutrients (*P* > 0.10; Table 3). Final BW, total weight gain (TWG), and ADG showed a positive linear effect response to increasing NFeed inclusion, with the highest values observed for the 100% NFeed diet (*P* ≤ 0.01; Table 4). For each 1% increase in NFeed inclusion in the diet, there was an estimated increase of 0.42126 kg in final BW, 0.46491 kg in TWG, and 0.00423 kg/day in overall ADG.

**Table 3:** Nutrient intake of Nellore young bulls fed diets with different levels of NFeed

	NFeed levels					SEM	*P*-value	
	0% NFeed		50% NFeed	75% NFeed	100% NFeed		L	Q
Intake, kg/day								
DM	12.6		12.8	12.8	12.9	0.21	0.13	0.90
OM	12.0		12.1	12.3	12.2	0.20	0.16	0.87
CP	2.0		2.0	2.0	2.0	0.04	0.19	0.87
EE	0.5		0.5	0.5	0.5	0.01	0.32	0.93
NDF	3.0		3.0	3.0	3.0	0.07	0.14	0.90
Intake, %BW								
DM	2.67		2.63	2.63	2.65	0.079	0.70	0.54
NDF	0.63		0.62	0.62	0.62	0.013	0.80	0.54

0% NFeed (100% of non-protein nitrogen from urea), 50% NFeed (50% of non-protein nitrogen from urea and 50% of non-protein nitrogen from NFeed), 75% NFeed (25% of non-protein nitrogen from urea and 75% of non-protein nitrogen from NFeed) and 100% NFeed (100% of non-protein nitrogen from NFeed)

L=first-degree regression; Q= second-degree regression; OM = organic matter, CP = crude protein; EE = ethereal extract; NNP = non-protein nitrogen; BW = body weight

**Table 4:** Performance and carcass characteristics of Nellore young bulls fed diets with different levels of NFeed

	NFeed levels				SEM	*P*- value	
	0% NFeed	50% NFeed	75% NFeed	100% NFeed		L	Q
Final body weight (kg)^1^	551.67b	578.50ab	589.83ab	591.80a	15.62	0.01	0.57
Total weight gain (kg)^2^	156.83b	184.00ab	195.17ab	202.40a	12.29	0.002	0.67
Average daily gain (kg/day)^4^	1.43b	1.67ab	1.77ab	1.84a	0.11	0.002	0.67
Feed conversion (kg/kg)^5^	8.23a	7.00b	6.75b	6.49b	0.71	0.003	0.43
Feed efficiency (kg/kg)^6^	0.12b	0.15ab	0.15ab	0.16a	0.01	0.003	0.60
Hot carcass weight (kg)	322.77	323.90	335.17	327.64	9.63	0.47	0.81
Dressing (%)^7^	58.50a	55.95bc	56.82b	55.34c	0.61	0.0004	0.42

*Means followed by a lowercase letter in the same row differ according to the Tukey test (*P* < 0.05)

0% NFeed (100% of non-protein nitrogen from urea), 50% NFeed (50% of non-protein nitrogen from urea and 50% of non-protein nitrogen from NFeed), 75% NFeed (25% of non-protein nitrogen from urea and 75% of non-protein nitrogen from NFeed) and 100% NFeed (100% of non-protein nitrogen from NFeed)

L= first-degree regression; Q = second-degree regression; TWG = total weight gain; ADG = average daily gain; HCW = hot carcass weight

*x* = Extruded urea enriched with essential oils level (NFeed)

^1^Y_final BW_ = 554.25429 + 0.42126*x* (R^2^ = 0.24; RMSE = 29.05)

^2^Y_TWG_ = 158.44857 + 0.46491*x* (R^2^ = 0.37; RMSE = 23.29)

^4^Y_ADG_ = 1.44044 + 0.00423*x* (R^2^ = 0.37; RMSE = 0.21)

^5^Y_Feed conversion_ = 8.10338-0.01756*x* (R^2^ = 0.33; RMSE = 0.97)

^6^Y_Feed efficiency_ = 0.12414 + 0.00034857*x* (R^2^ = 0.34; RMSE = 0.02)

^7^Y_dressing_ = 58.23610-0.02815*x* (R^2^ = 0.44; RMSE = 1.22)

Feed efficiency and feed conversion have a linear effect (*P* = 0.003), with the 100% NFeed diet resulting in the greatest feed efficiency and the lowest feed conversion. According to the regression equations, each 1% increase in NFeed inclusion promoted an estimated decrease of 0.01756 units in feed conversion and an increase of 0.00034857 units in feed efficiency. There was no effect on hot carcass weight (*P* = 0.47). However, dressing percentage decreased linearly as NFeed inclusion increased, with an estimated reduction of 0.02815% for each 1% increase in NFeed inclusion in the diet.

When comparing the means of performance variables, the replacement of urea with 100% NFeed resulted in a significant increase (*P* < 0.05) of 40.13 kg in final body weight, 45.57 kg in total weight gain, 0.41 kg/day in average daily gain, and 0.0242 in feed efficiency. Conversely, there was a significant reduction (*P* < 0.05) of 2.27 in feed conversion and 3.16% in carcass yield among animals receiving 100% NFeed as the non-protein nitrogen (NPN) source in the diet (Table 4).

**Fig. 1:**
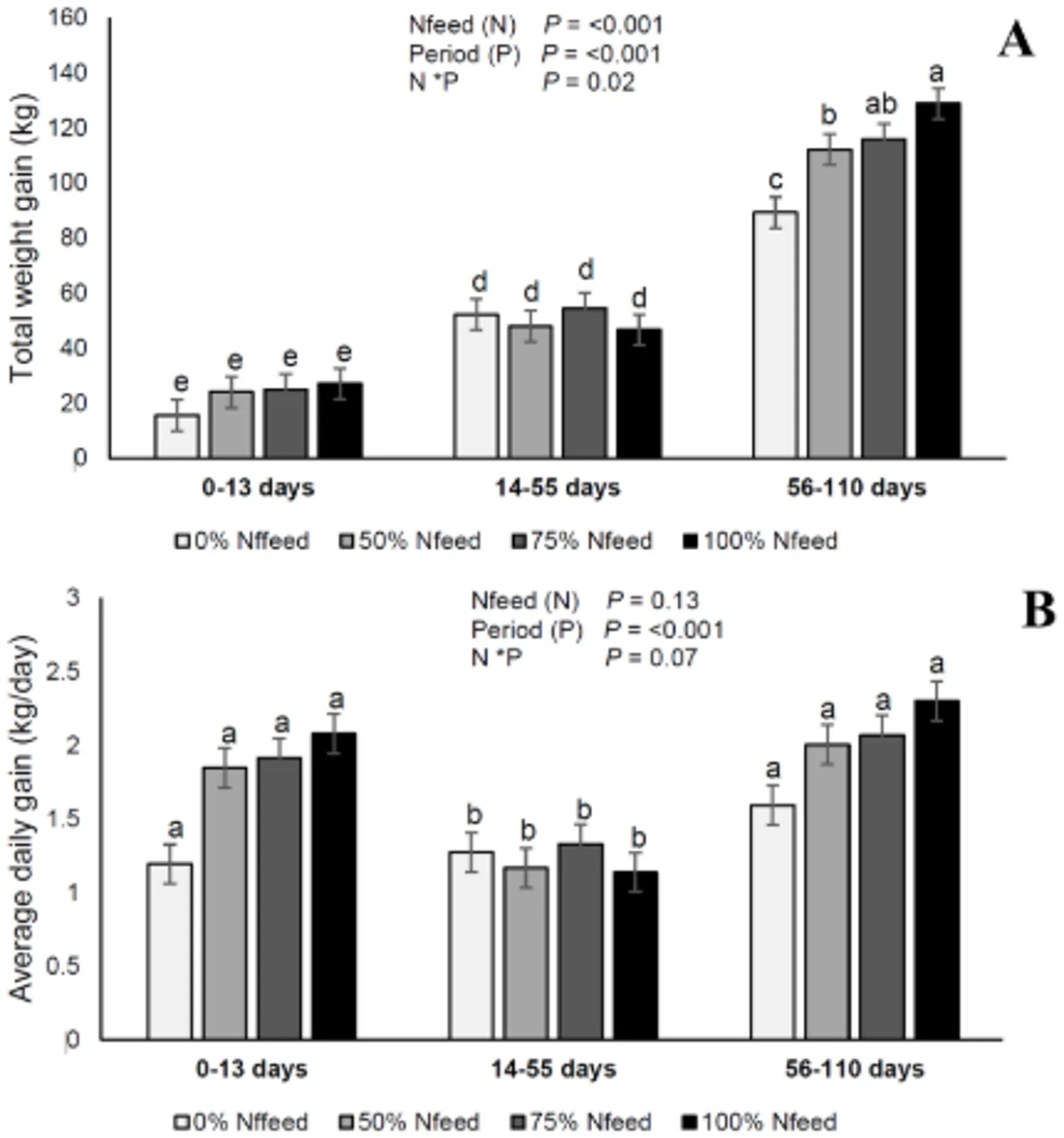
Total weight gain (**A**) and Average daily gain (**B**) of Nellore young bulls fed diets with different levels of NFeed at different periods

Considering the feedlot periods (Fig. 1), there was an NFeed × period interaction for TWG (Fig. 1A; *P* = 0.02). The TWG was lower in the first 13 days of the trial (adaptation phase), and did not differ among NFeed treatments. However, during the final period (56–110 days), animals fed 100% NFeed had the greatest TWG (128.8 kg), whereas those fed 0% NFeed had the lowest gain (89.2 kg).

For ADG, there was no effect of NFeed × period (Fig. 1B; *P* = 0.07), but ADG was influenced by the feedlot period (*P* < 0.001). Regardless of treatment, ADG was greater during the adaptation (0–13 days) and finishing (56–110 days) periods, averaging 1.77 and 1.99 kg/day, respectively, and the lowest ADG was observed during the intermediate period (14–55 days), with an average of 1.22 kg/day.

There was a quadratic effect of NFeed inclusion plasma urea, and gamma glutamyl transferase (GGT) (*P* < 0.05; Table 5). Plasma urea and GGT showed minimum estimated values at approximately 64.49% and 59.2% NFeed inclusion, corresponding to 21.84 mg/dL and 10.95 U/L, respectively. When comparing the means of blood parameters, the replacement of urea with 50% NFeed resulted in lower results (*P* < 0.05) in plasma urea and GGT averages (Table 5). There was no effect of the other blood parameters for the different levels of NFeed replacement in the diets of young bulls. A negative correlation between plasma urea and final BW, ADG, and TWG was detected by correlation analysis. Likewise, Aspartate aminotransferase was negatively correlated with ADG and total gain and glucose had negative correlation with final BW (Table 6).

**Table 5:** Blood parameters of Nellore young bulls fed diets with different levels of NFeed

		NFeed levels				SEM	*P*-value	
		0% NFeed	50% NFeed	75% NFeed	100% NFeed		L	Q
Plasma compounds								
Total protein (g/dL)		4.7	3.9	4.2	5.8	0.67	0.13	0.07
Albumin (g/dL)		2.6	2.1	2.3	2.9	0.34	0.14	0.11
Creatinine (mg/dL)		1.7	1.4	1.5	1.6	0.18	0.21	0.23
Cholesterol (mg/dL)		89.9	69.4	80.1	102.5	12.97	0.12	0.08
Triglycerides (mg/dL)		17.9	12.0	13.1	13.2	3.44	0.29	0.43
Urea (mg/dL)^1^		33.3a	21.6b	23.2ab	24.8ab	2.69	0.01	0.03
Glucose (mg/dL)		91.1	79.2	81.1	71.8	8.51	0.64	0.98
Enzymes								
ALP (U/L)		17.9	14.2	14.4	16.9	2.14	0.15	0.19
AST (U/L)		103.2	73.2	83.8	88.8	8.09	0.27	0.18
GGT (U/L)^2^		25.1a	10.4b	13.2ab	17.1ab	3.67	0.01	0.02

*Means followed by a lowercase letter in the same row differ according to the Tukey test (*P* < 0.05)

0% NFeed (100% of non-protein nitrogen from urea), 50% NFeed (50% of non-protein nitrogen from urea and 50% of non-protein nitrogen from NFeed), 75% NFeed (25% of non-protein nitrogen from urea and 75% of non-protein nitrogen from NFeed) and 100% NFeed (100% of non-protein nitrogen from NFeed)

L= first-degree regression; Q = second-degree regression; ALP = Alkaline phosphatase; AST = Aspartate aminotransferase; GGT = Gamma glutamyl transferase

*x* = Extruded urea enriched with essential oils level (NFeed)

^1^Y _Urea_ = 33.15242-0.35080*x* + 0.00272* × *^2^ (R^2^ = 0.36; RMSE = 6.47)

^2^Y _GGT_ = 24.98247-0.47436*x* + 0.00401* × *^2^ (R^2^ = 0.31; RMSE = 0.32)

**Table 6:** Correlation coefficients among blood parameters and performance traits of Nellore young bulls fed diets with different levels of NFeed

	Total protein	Albumin	Creatinine	CHOL	TRIG	ALP	AST	GGT	UREA	GLUC
DMI	-0.043	-0.113	0.025	-0.065	-0.025	-0.017	0.024	0.060	-0.218	-0.089
ADG	-0.188	-0.269	-0.245	-0.239	-0.339	-0.228	-0.390*	-0.216	-0.431**	-0.218
TWG	-0.188	-0.269	-0.246	-0.240	-0.342	-0.228	-0.390*	-0.217	-0.433**	-0.222
Final BW	-0.219	-0.281	-0.069	-0.116	-0.336	-0.229	-0.030	-0.052	-0.377*	-0.372*

0% NFeed (100% of non-protein nitrogen from urea), 50% NFeed (50% of non-protein nitrogen from urea and 50% of non-protein nitrogen from NFeed), 75% NFeed (25% of non-protein nitrogen from urea and 75% of non-protein nitrogen from NFeed) and 100% NFeed (100% of non-protein nitrogen from NFeed)

CHOL = Cholesterol; TRIG = Triglycerides; ALP = Alkaline phosphatase; AST = Aspartate aminotransferase; GGT = Gamma glutamyl transferase; GLU = glucose; DMI = Dry matter intake; ADG = average daily gain; TWG = total weight gain; BW= body weight

** *P* ≤ 0.05; *0.05 < *P* ≤ 0.10

When comparing the means of economic variables, replacing urea with NFeed had no significant effect (*P* > 0.05) on revenue per animal (US$937.55/animal), margin per animal (US$601.40/animal), margin per animal/day (US$6.00/animal/day), or margin per kg of carcass (US$1.84/kg carcass equivalent). On the other hand, the 100% NFeed treatment showed an increase in cost per animal and per day compared to the treatment without NFeed (0% NFeed); however, there was a significant reduction in the cost per unit of gain (US$0.42/day). The daily and total feed costs increased linearly with increasing NFeed inclusion, with the highest costs observed for the 100% NFeed diet (*P* < 0.0001). For each 1% increase in NFeed inclusion, daily and total costs increased by an estimated 0.00169 US$/animal/day and 0.17155 US$/animal, respectively. On the other hand, the cost of gain exhibited a linear decrease with increasing levels of NFeed inclusion, with an estimated reduction of 0.00421 units for each 1% increase in NFeed inclusion (*P* = 0.01; Table 7).

**Table 7:** Economic performance of Nellore young bulls fed diets with different levels of NFeed

	NFeed levels				SEM	*P*-value	
	0% NFeed	50% NFeed	75% NFeed	100% NFeed		L	Q
Revenue (US$/animal)	924.40	927.60	959.90	938.30	27.59	0.47	0.81
*Costs*							
daily (US$/animal/day)^1^	3.27c	3.33bc	3.40ab	3.44a	0.03	0.0001	0.59
total (US$/animal)^2^	327.20c	333.26bc	340.16ab	343.95a	3.36	0.0001	0.60
gain (US$/Kg gain)^3^	2.14a	1.82ab	1.78ab	1.72b	0.07	0.01	0.45
Total margin (US$/animal)	597.2	594.4	619.7	594.4	27.59	0.85	0.75
Margin per animal (US$/animal/day)	5.97	5.94	6.20	5.90	0.26	0.85	0.76
Margin per carcass (US$/kg equivalent carcass)	1.85	1.84	1.85	1.81	0.18	0.66	0.78

*Means followed by a lowercase letter in the same row differ according to the Tukey test (*P* < 0.05)

0% NFeed (100% of non-protein nitrogen from urea), 50% NFeed (50% of non-protein nitrogen from urea and 50% of non-protein nitrogen from NFeed), 75% NFeed (25% of non-protein nitrogen from urea and 75% of non-protein nitrogen from NFeed) and 100% NFeed (100% of non-protein nitrogen from NFeed)

L= first-degree regression; Q = second-degree regression

*x* = Extruded urea enriched with essential oils level (NFeed)

^1^Y_cost daily_ =3.26600 + 0.00169*x* (R^2^ = 0.53; RMSE = 0.06)

^2^Y_cost total_ =326.49195 + 0.17155*x* (R^2^ = 0.53; RMSE = 6.19)

^3^Y_cost gain_ =2.10157-0.00421*x* (R^2^ = 0.29; RMSE = 0.25)

## Discussion

The potential for urea to induce ammonia toxicity was assessed through blood parameters, with urea concentrations serving as key biomarkers. In this study, despite plasma urea levels showing a quadratic response, reaching the lowest concentration observed at approximately 58% replacement, plasma urea concentrations remained within the physiological reference range (20–30 mg/dL) (Kaneko et al. 2008) for all treatments, except those receiving diets with 100% urea as the non-protein source. This may reflect an asynchrony between the rapid release of ammonia from urea hydrolysis and the availability of fermentable energy in the rumen (Calsamiglia et al. 2010). When ammonia release exceeds microbial assimilation capacity, excess nitrogen is absorbed through the rumen wall, converted into urea in the liver, and subsequently detected as increased plasma urea concentration (Huntington and Archibeque 2000). This mechanism impairing microbial protein synthesis, consequently, reduced microbial protein outflow may have limited metabolizable protein supply, contributing to the observed reductions in ADG and final BW in animals fed 100% urea diet. A negative correlation between plasma urea concentration and performance traits supports this interpretation. According to Owens et al. (2014), excessive ruminal ammonia accumulation increases the energetic cost of hepatic detoxification and reduces overall nitrogen-use efficiency. Supporting the present results, Ítavo et al. (2016) evaluating sources of non-protein nitrogen, found that extruded urea had slow solubility time compared to livestock urea, which reflected in high ADG, and carcass gain in Nellore steers in deferred pasture.

Gamma-glutamyl transferase (GGT), a key biomarker of hepatic function and liver damage (Kaneko et al. 2008), was high in animals fed 100% urea diet. In this treatment (100% urea diet), GGT levels exceeded the physiological reference range of 6.1 to 17.4 U/L (Kaneko et al. 2008), indicating potential liver injury. Chronic urea toxicity can lead to excessive hepatic nitrogen metabolism, which is often reflected by increased serum GGT activity (Salami et al. 2021). Furthermore, elevated blood ammonia concentrations may increase oxidative stress and inflammatory responses in hepatic tissue, which could partially explain the reduced productive performance observed in animals fed conventional urea exclusively. The simultaneous increase in plasma urea and GGT concentrations in animals fed the 100% urea diet suggests greater hepatic metabolic demand associated with less efficient nitrogen utilization.

On the other hand, animals fed diets with partial or complete replacement by NFeed maintained GGT concentrations within or below the reference range. This suggests that NFeed inclusion may mitigate hepatic stress. This may be related not only to the slower release characteristics of extruded urea, but also the ability of essential oils to support metabolic processes without compromising liver health, thereby enhancing liver functionality and contributing to improved animal performance (Ghosh et al. 2010). Essential oils have been reported to modulate ruminal fermentation, reduce nitrogen and energy losses, boost nitrogen retention, and reduce methane emissions (Ferreira et al. 2024; García-Rodríguez et al. 2024).

The greater ADG observed during the adaptation period may be explained by compensatory growth, a common response of cattle transitioning from grazing systems to high-concentrate feedlot diets. Following a period of lower nutrient availability on pasture, animals often exhibit enhanced growth rates due to improved efficiency of nutrient utilization and tissue accretion. On the other hand, the reduction in performance observed during the subsequent period may have been influenced by rainfall events recorded during the experiment.

Animals fed the diet containing 100% NFeed diet had greater ADG, TWG, and final BW. These results indicate that the inclusion of essential oils may enhance ruminal fermentation efficiency and, consequently, improve animal performance (Ferreira et al. 2024). NFeed is a commercial nitrogen supplement which is composed of extruded urea enriched with garlic and cinnamon essential oils (Oliveira et al. 2025). Garlic and cinnamon have been shown to enhance nutrient digestibility and exhibit antimicrobial and antioxidant properties (Besharati et al. 2023; Ding et al. 2023). Recent studies indicate that essential oils from garlic and cinnamon increase propionate production, reduce the acetate: propionate ratio, and methane emissions. Greater propionate production enhances glucose synthesis through hepatic gluconeogenesis, thereby increasing the availability of energy for muscle deposition and growth from fermented substrates, promoting greater weight gain and better feed conversion (Ghosh et al. 2010; Khorrami et al. 2015; García-Rodríguez et al. 2024). Additionally, these results suggest that the effects of feed additives on performance are dose-dependent, and that low inclusion levels may be insufficient to fully activate their beneficial responses (Liu et al. 2026).

Regarding the economic performance, although no differences were observed in revenue, and high daily and total costs for 100% NFeed due to product cost, the lower cost per gain in animals fed 100% NFeed diet was due to their higher average daily gain. Silva et al. (2025) evaluated the effect of NNP (extruded urea vs. livestock urea plus protected urea) sources added to the total ration on the economic performance of cull heifers finished in a feedlot, and found no difference in revenue and costs of gain between NNP sources. Our results are lower than the 2.40 US$/kg gain shown by these authors. Ítavo et al. (2023) reported margins per animal ranging from US$ 433 to US$ 442 when evaluating extruded urea levels of 50 to 80 g/100 kg of body weight in the diets of feedlot cattle. These values are lower than the margins per animal observed in the present study, likely due to differences in the roughage-to-concentrate ratio and annual fluctuations in concentrate costs.

Despite all adjusted regression equations of productive performance as a function of NFeed levels show a linear increase in variables (final BW, total weight gain, ADG, and feed efficiency). Furthermore, the feed conversion equation confirm the improvement in the transformation of diet in performance with linear negative equation (Table 4). However, the plasma urea concentration show an optimal NFeed level at 58% of replacement of urea. This way, when using this maximal level in the equations Thus, our results of economic performance, a decrease in daily costs of 0.07 US$/animal/day, and, consequently, in total costs of 7.21 US$/animal.

## Conclusion

The replacement of conventional urea with extruded urea enriched with essential oils (NFeed) represents a viable nutritional strategy for finishing beef cattle in feedlot systems. Increasing levels of NFeed linearly improved animal performance while maintaining similar feed intake, increasing feed efficiency reducing feed conversion, and the cost per kilogram of body weight gain. Thus, we recommend a partial replacement of urea by NFeed as a non-protein source in beef cattle feedlot.

## Data Availability

The datasets generated and/or analyzed during the current study are available from the corresponding author in reasonable request.
